# Plasma proenkephalin A and incident chronic kidney disease and albuminuria in the REasons for Geographic And Racial Differences in Stroke (REGARDS) cohort

**DOI:** 10.1186/s12882-023-03432-7

**Published:** 2024-01-10

**Authors:** Alexander L. Bullen, Ronit Katz, Sayna Poursadrolah, Samuel A. P. Short, D. Leann Long, Katharine L. Cheung, Shilpa Sharma, Tala Al-Rousan, Alma Fregoso, Janin Schulte, Orlando M. Gutierrez, Michael G. Shlipak, Mary Cushman, Joachim H. Ix, Dena E. Rifkin

**Affiliations:** 1 Nephrology Section, Veterans Affairs San Diego Healthcare System, La Jolla, CA USA; 2 Division of Nephrology and Hypertension, Department of Medicine, University of California San Diego, San Diego, CA USA; 3 University of Washington, Seattle, WA USA; 4 Department of Medicine, University of California San Diego, San Diego, CA USA; 5 University of North Carolina, Chapel Hill, North Carolina USA; 6 Department of Biostatistics, School of Public Health, University of Alabama at Birmingham, Birmingham, AL USA; 7 Division of Nephrology, Larner College of Medicine, University of Vermont, Burlington, VT USA; 8 Division of Nephrology, David Geffen School of Medicine at UCLA, Los Angeles, CA USA; 9 Nephrology Section, Veteran Affairs Greater Los Angeles Healthcare System, Los Angeles, CA USA; 10 Division of Preventive Medicine, Department of Family Medicine and Public Health, University of California San Diego, San Diego, CA USA; 11 School of Medicine, University of California San Diego, San Diego, CA USA; 12 SphingoTec GmbH, Hennigsdorf, Germany; 13 Division of Nephrology, Department of Medicine, University of Alabama at Birmingham, Birmingham, AL USA; 14 Kidney Health Research Collaborative, Department of Medicine, University of California, San Francisco, CA USA; 15 Department of Medicine, San Francisco VA Medical Center, San Francisco, CA USA; 16 Department of Pathology and Laboratory Medicine, Larner College of Medicine, University of Vermont, Burlington, VT USA; 17 Department of Medicine, Larner College of Medicine, University of Vermont, Burlington, VT USA

**Keywords:** biomarker, proenkephalin A, chronic kidney disease, albuminuria

## Abstract

**Background:**

Plasma proenkephalin A (PENK-A) is a precursor of active enkephalins. Higher blood concentrations have been associated with estimated glomerular filtration rate (eGFR) decline in European populations. Due to the significant disparity in incident chronic kidney disease (CKD) between White and Black people, we evaluated the association of PENK-A with incident CKD and other kidney outcomes among a biracial cohort in the U.S.

**Methods:**

In a nested cohort of 4,400 participants among the REasons for Geographic And Racial Differences in Stroke, we determined the association between baseline PENK-A concentration and incident CKD using the creatinine-cystatin C CKD-EPI 2021 equation without race coefficient, significant eGFR decline, and incident albuminuria between baseline and a follow-up visit 9.4 years later. We tested for race and sex interactions. We used inverse probability sampling weights to account for the sampling design.

**Results:**

At baseline, mean (SD) age was 64 (8) years, 49% were women, and 52% were Black participants. 8.5% developed CKD, 21% experienced ≥ 30% decline in eGFR and 18% developed albuminuria. There was no association between PENK-A and incident CKD and no difference by race or sex. However, higher PENK-A was associated with increased odds of progressive eGFR decline (OR: 1.12; 95% CI 1.00, 1.25). Higher PENK-A concentration was strongly associated with incident albuminuria among patients without diabetes mellitus (OR:

1.29; 95% CI 1.09, 1.53).

**Conclusion:**

While PENK-A was not associated with incident CKD, its associations with progression of CKD and incident albuminuria, among patients without diabetes, suggest that it might be a useful tool in the evaluation of kidney disease among White and Black patients.

## Introduction

Chronic kidney disease (CKD) is common, costly, and associated with cardiovascular disease and mortality [1–3]. Current screening methods are suboptimal because serum creatinine is insensitive to early reductions in GFR [4], and nearly 75 percent of persons with CKD stage 3 do not have albuminuria [5, 6]. Enkephalins are endogenous opioids produced throughout the body, including the kidneys, that act primarily in delta-opioid receptors [7, 8]. These opioid receptors are found in high numbers in the kidneys, second only to the central nervous system [9]. Although enkephalins in the kidneys may inhibit antidiuretic hormone [10] and induce diuresis and natriuresis [11], their physiologic role is not yet fully understood. The biologically mature active enkephalin peptides (methionine-enkephalin, leucine-enkephalin) have a half-life in human plasma of fewer than 15 min and are therefore difficult to measure reliably [12, 13].

Plasma proenkephalin A (PENK-A) is a 4.5 kDa molecule and is a precursor of enkephalins [14]. PENK-A is freely filtered through the glomerulus; it is not secreted or protein-bound and it is a putative marker of glomerular filtration [15]. However, it may also reflect other aspects of kidney health [16]. While our understanding of its association with CKD development is limited, two prior studies reported that higher concentration of PENK-A was associated with incident CKD [16, 17]. One of these studies utilized Mendelian randomization and suggested a causal role of PENK-A in CKD development [16]. However, these prior studies evaluated the association between PENK-A and incident CKD in relatively homogenous European populations. To our knowledge, the relationship of PENK-A with incident CKD and albuminuria in different race groups has not been studied.

Since PENK-A may be an emerging marker of incident CKD, and since persons of African ancestry have both high risk and unique pathways for CKD progression relative to individuals of European ancestry, such as APOL-1 nephropathy, we sought to determine the relative strength of association of PENK-A with incident CKD by race and to evaluate its association with incident albuminuria [18]. We hypothesized that PENK-A would be associated with each outcome and that these associations might differ between Black and White participants.

## Methods

### Study design and participants

The REasons for Geographic And Racial Differences in Stroke (REGARDS) is a population-based cohort of individuals aged ≥ 45 years designed to study the reasons for the higher stroke mortality noted among Black versus White adults and among adults residing in the Southeast region of the United States [19, 20]. A total of 30,239 adults were recruited between January 2003 and June 2007. Among the exclusion criteria were race other than Black or White, active treatment of cancer, medical conditions that would prevent long-term participation, residence in or inclusion on a waiting list for a nursing home, or inability to communicate in English. Potential participants were contacted by mail with a subsequent computer-assisted telephone interview, then an in-home visit for a physical exam and blood collection followed. The institutional review boards of the participating institutions approved the REGARDS study, and all participants provided verbal consent before the telephone interview and written informed consent before completing the in-home study visit. Participants or their proxies were contacted every six months by telephone to assess outcomes, including death. Approximately ten years after the baseline visit, a single follow-up visit and extensive telephone interview was conducted using the same methods. Details of the study design have been previously described [19].

According to REGARDS policy, the aims and analysis plan for this manuscript were prespecified and reviewed and approved by the REGARDS publications committee, which also reviewed the final manuscript and assured the a priori plans were followed.

**Fig. 1:**
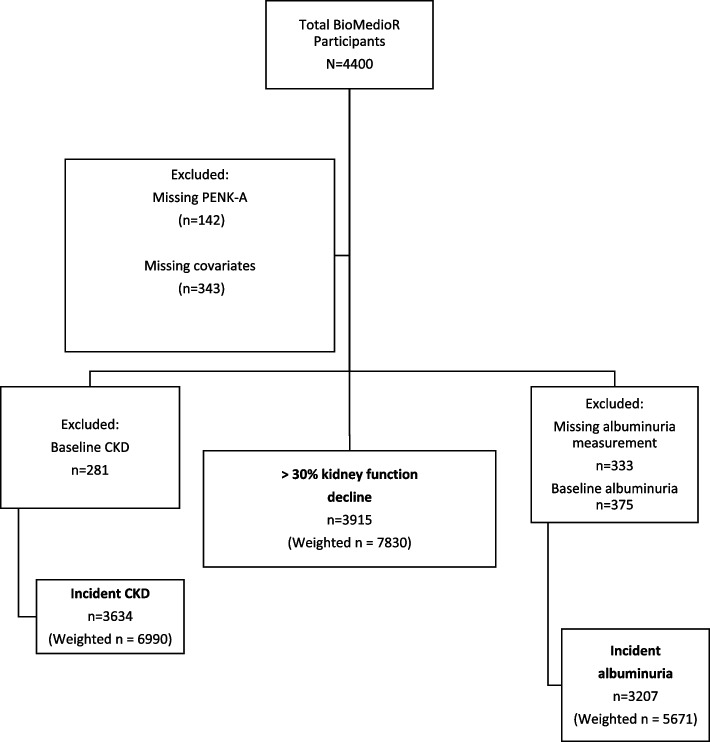
Flowchart of BioMedioR participants for analysis. Abbreviation: PENK-A, proenkephalin A; eGFR: estimated glomerular filtration rate; CKD, chronic kidney disease

Our analytic cohort included all REGARDS participants who were part of a nested cohort study within REGARDS named Biomarker Mediators of Racial Disparities in Risk Factors (BioMedioR) [21]. BioMedioR is studying the role of biomarkers in understanding racial differences in incident hypertension and diabetes [21]. This nested cohort included 4,400 individuals who completed the second visit and had information on hypertension and diabetes status at baseline and follow-up (13,912), deliberately sampled to obtain equal groups based on race and sex. In this study, 142 participants were excluded due to missing PENK-A at baseline and 343 participants due to missing covariates (Fig. 1). The final analytic sample size was 3,915 (weighted *n* = 7830).

### Exposure variable

Morning blood specimens were collected at baseline, shipped overnight to a central laboratory at the University of Vermont [22], and stored at -80 °C without a prior thaw until PENK-A measurement, consistent with a prior study [16]. Proenkephalin A 119–159 was measured in duplicate in EDTA plasma samples using the immunoluminometric Sphingotest® assay (SphingoTec GmbH, Hennigsdorf, Germany) as described previously [15, 23]. Results were averaged. The laboratory performing the biomarker measurement was blinded to clinical and demographic data of the patients. The analytical coefficient of variation range was 0.6–7.4%. PENK-A was analyzed using both raw quartiles and continuously per standard deviation.

### Covariates

Information on baseline age, race, sex, smoking status, prevalent coronary artery disease, lipid-lowering medication use, and antihypertensive medication use was collected by self-report during the telephone interview. Height and weight were measured at the in-home visit, and body mass index (BMI) was calculated as weight (kg)/height (m^2^).

Systolic (SBP) and diastolic blood pressure (DBP) were measured by trained personnel three times after 5 min of rest, and the averages of the second and third measures were recorded. Hypertension was defined as SBP ≥ 140 mmHg, DBP > 90 mmHg, or self-reported use of antihypertensive medications. Diabetes was defined as fasting glucose ≥ 126 mg/dL, non-fasting glucose ≥ 200 mg/dL, or using either oral hypoglycemic medications or insulin. Serum creatinine was calibrated to an international isotope dilution mass spectroscopic (IDMS)-traceable standard, measured by colorimetric reflectance spectrophotometry (Ortho Vitros Clinical Chemistry System 950IRC, Johnson & Johnson Clinical Diagnostics, www.orthochemical.com). Serum cystatin C was measured with high sensitivity particle-enhanced immunonephelometry (N Latex Cystatin C on the BNII, Dade Behring, Deerfield, IL). The estimated glomerular filtration rate (eGFR) was calculated using the 2021 CKD-EPI creatinine- and cystatin C-based equation without a race coefficient [24].

Urine albumin and creatinine were measured using the random spot urine specimen by nephelometry (BN ProSpec Nephelometer, Dade Behring, Marburg, Germany) and Modular-P chemistry analyzer (Roche/Hitachi, Indianapolis, IN), respectively. Spot urinary albumin-to-creatinine ratio (uACR) was calculated in mg/g.

### Outcomes

The outcomes were incident CKD at the second visit, defined as eGFR < 60 mL/min/1.73m^2^ and at least 40% decline in individuals with baseline eGFR > 60 mL/min/1.73m^2^, progressive eGFR decline, defined as a > 30% decrease in eGFR (where we did not exclude participants with baseline CKD), and incident albuminuria, defined as new uACR ≥ 30 mg/g among participants with baseline uACR < 30 mg/g. In sensitivity analyses, we also evaluated the association between PENK-A and incident CKD defined as eGFR < 60 ml/min/1.73m^2^ and at least 25% decline in individuals with baseline eGFR > 60 ml/min/1.73m^2^ [23].

### Statistical analyses

We first examined the distribution of demographics and risk factors for incident CKD among PENK-A quartiles. We then evaluated the correlation between PENK-A and cystatin C and creatinine. Inverse probability sampling weights (IPSW) were used to account for the BioMedioR sampling design [21]. All the statistical analyses were done with weights. Since there were only two visits 9.4 years apart, we used logistic regression rather than time-to-event models. Sequential nested models were fit for each outcome. Covariates for multivariable models were selected a priori based on biological plausibility. Model 1 adjusted for age, sex, and race. Model 2 additionally adjusted for BMI, SBP, use of hypertensive medications, diabetes, smoking, total cholesterol, and a history of cardiovascular disease. Model 3 additionally adjusted for baseline eGFR and uACR. We tested for interactions of PENK-A with race and sex in the fully adjusted model. For incident albuminuria, we also evaluated the interaction between PENK-A and diabetes mellitus.

All analyses were conducted using STATA/PC version 16.1 (StataCorp LLC, College Station, TX) and R version 4.1.1 (https://www.R-project.org/). *P* values < 0.05 were considered statistically significant for all analyses, except for interaction terms where a *P* value of < 0.10 was considered significant.

## Results

### Baseline characteristics

**Fig. 2:**
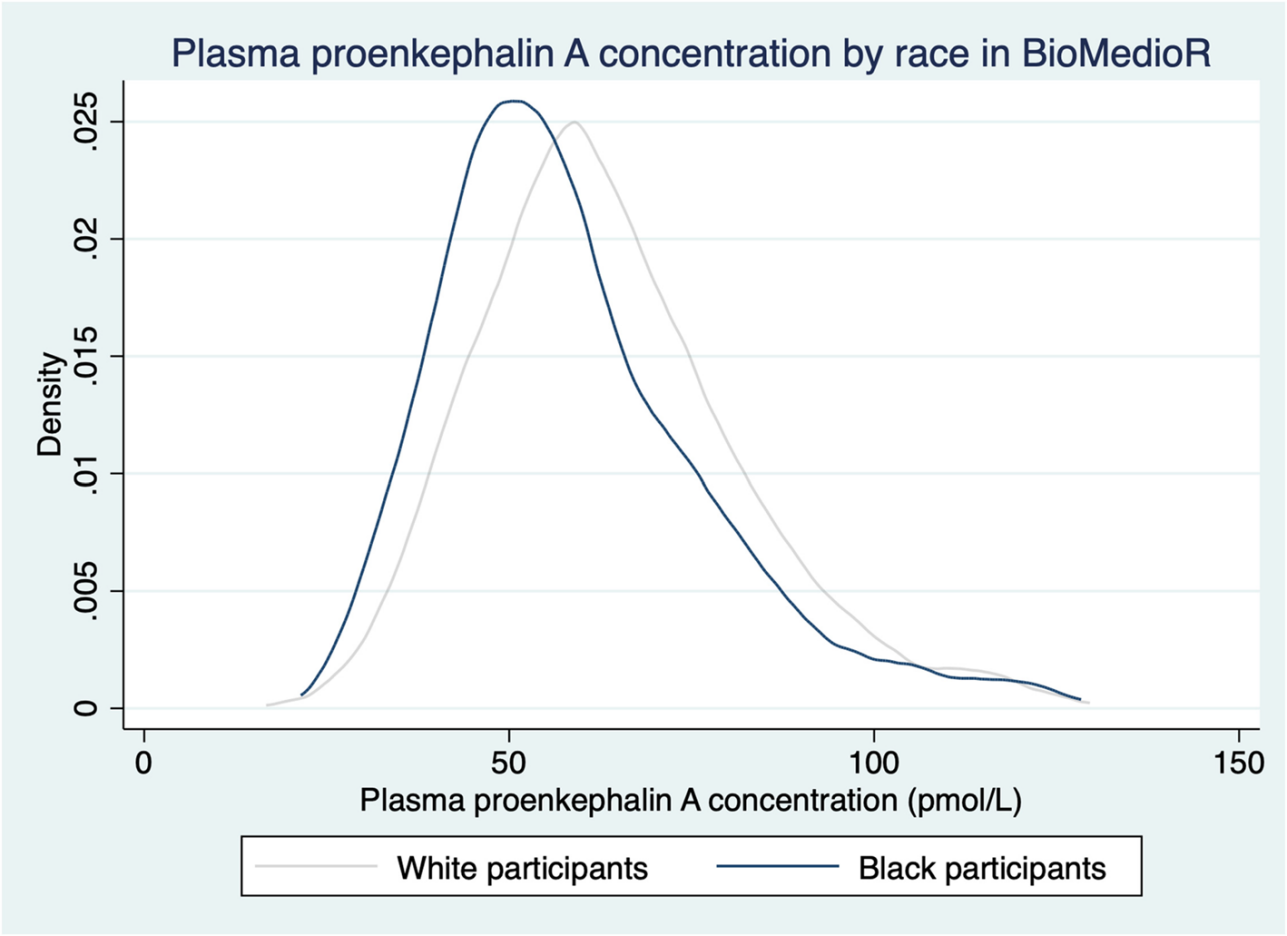
Plasma proenkephalin-A concentration by race in BioMedioR. *n* = 7818. Abbreviation: PENK-A, proenkephalin A

**Fig. 3:**
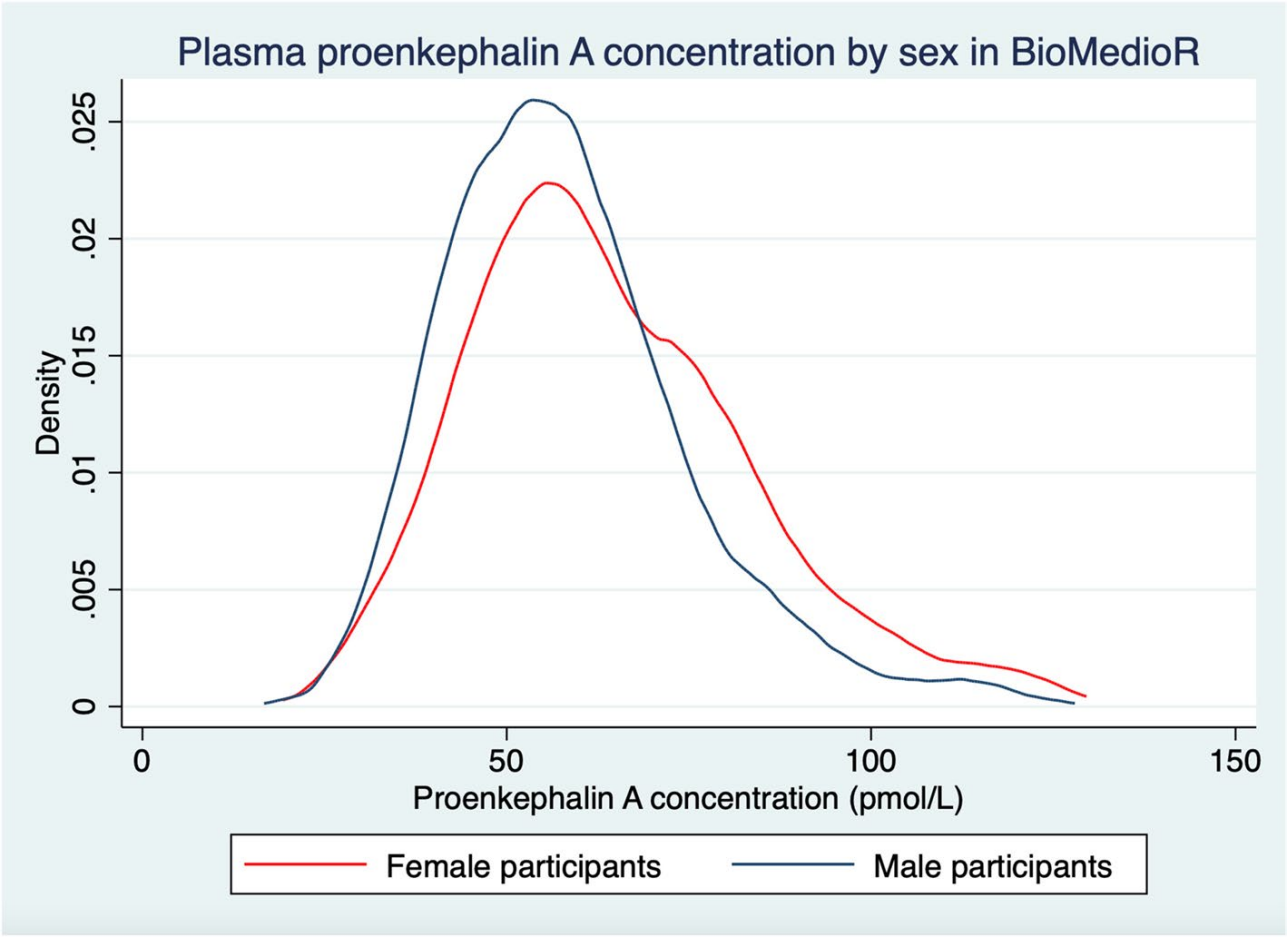
Plasma proenkephalin-A concentration by sex in BioMedioR. *n* = 7818. Abbreviation: PENK-A, proenkephalin A

Among 7830 participants, the mean (SD) age was 64 (8) years, 49% were women, and 52% were Black. Median baseline eGFR (interquartile range [IQR]) was 87 (73, 99) mL/min/1.73m^2^. The correlation between PENK-A and cystatin C was 0.72 and between PENK-A and serum creatinine was 0.67. The median PENK-A concentration (IQR) was 59.5 pmol/L (48.9, 73.9 pmol/L). Median PENK-A was higher among White (62.6; IQR 52.2, 77 pmol/L) than Black (56.2; IQR 46.7, 70.1 pmol/L) participants, Fig. 2. Females had higher median PENK-A (61.7; IQR 50.5, 77.3 pmol/L) than males (57.4; IQR 47.1, 69.7 pmol/L), Fig. 3. Baseline characteristics by quartile of PENK-A are shown in Table 1. Compared to participants with lower PENK-A at baseline, participants with higher PENK-A concentrations were more likely to be older, were less likely to have diabetes, and had higher albuminuria and lower eGFR levels.

**Table 1:** Baseline characteristics of BioMedioR participants by PENK-A quartiles^a^, weighted

PENK-A quartile	Q1	Q2	Q3	Q4
PENK-A range, pmol/L	< 49	49–59	60–74	> 74
Sample size, unweighted	1032	988	996	899
Weighted N	1958	1958	1957	1957
Age, years (SD)	62 (7)	63 (8)	65 (8)	67 (8)
Female, n (%)	803 (41)	920 (47)	939 (48)	1134 (58)
Black, n (%)	1234 (63)	1116 (57)	900 (46)	861 (44)
Body mass index, kg/m^2^ (SD)	32 (6)	31 (6)	29 (5)	28 (5)
Diabetes mellitus, n (%)	705 (36)	627 (32)	430 (22)	528 (27)
Cardiovascular disease, n (%)	313 (16)	352 (18)	274 (14)	372 (19)
Systolic blood pressure, n (SD)	131 (16)	128 (16)	130 (17)	128 (17)
Diastolic blood pressure, n (SD)	79 (9)	78 (9)	77 (10)	75 (9)
Current tobacco use, n (%)	274 (14)	196 (10)	235 (12)	156 (8)
Serum creatinine, mg/dL (IQR)	0.79 (0.70, 0.92)	0.80 (0.70, 0.98)	0.89 (0.73, 0.98)	0.98 (0.79, 1.17)
Median serum cystatin C, mg/dL (IQR)	0.86 (0.76, 0.98)	0.89 (0.82, 1.03)	0.95 (0.85, 1.05)	1.09 (0.96, 1.31)
Median eGFR, ml/min/1.73m2 (IQR)^b^	98 (86, 107)	92 (81, 100)	87 (77, 96)	71 (56, 83)
Median uACR, mg/g (IQR)	8 (5, 22)	8 (4, 17)	7 (4, 15)	9 (5, 32)

*Abbreviations*: *eGFR* estimated glomerular filtration rate, u*ACR* albumin-to-creatinine ratio

^a^Weighted to parent cohort and excluding participants missing PENK-A

^b^estimated eGFR using the 2021 CKD-EPI creatinine- and cystatin-C based equation without race equation

### Relationship of PENK-A with incident CKD

We excluded 840 (10.7%) participants with prevalent CKD at baseline for the incident CKD analyses, leaving 6990 participants. Table 2 shows the relationship between PENK-A and incident CKD. During a mean follow-up of 9.4 years, 1728 (24.7%) developed incident CKD. When modeled continuously, each SD higher PENK-A concentration was not significantly associated with incident CKD across the sequence of adjusted models when all participants were evaluated together (OR for fully adjusted model: 1.02; 95% CI 0.88, 1.18).

**Table 2:** Association of PENK-A with incident CKD (defined as new eGFR < 60 mL/min/1.73m^2^ plus at least 40% eGFR decline)^a^

	**Quartiles of PENK-A**					
	**Q1**	**Q2**	**Q3**	**Q4**	**Per SD higher**	***P* value**^+^
	(OR, 95% CI)	(OR, 95% CI)	(OR, 95% CI)	(OR, 95% CI)	(OR, 95% CI)	
Range of PENK-A	< 48	49–57	58–70	> 71		
Number of events/Number at Risk (%)						
BioMedioR	78/909 (8.6)	79/908 (8.7)	75/909 (8.2)	77/908 (8.5)	309/3634 (8.5)	
Weighted	436/1747 (25.0)	436/1752 (24.9)	425/1744 (24.4)	431/1747 (24.7)	1728/6990 (24.7)	
Model 1*	Reference	0.95 (0.69, 1.32)	0.89 (0.63, 1.24)	0.86 (0.60, 1.23)	0.94 (0.82, 1.08)	0.42
Model 2**	Reference	1.14 (0.81, 1.61)	1.19 (0.84, 1.70)	1.31 (0.90, 1.91)	1.11 (0.96, 1.28)	0.15
Model 3***	Reference	1.10 (0.77, 1.57)	1.11 (0.76, 1.61)	1.02 (0.67, 1.55)	1.02 (0.86, 1.21)	0.82

P for interaction for sex: 0.14

P for interaction for race: 0.48

^a^ Weighted to parent cohort and excluding participants missing PENK-A and baseline CKD

^*^Adjusted for age, sex, race

^**^Adjusted for Model 1 plus body mass index, current smoking, coronary artery disease, systolic blood pressure, use of antihypertensive meds, diabetes mellitus

^***^Adjusted for Model 2 plus baseline eGFR and uACR

^+^ *P* value for SD higher of PENK-A

There was no significant interaction between PENK-A and race for the incident CKD outcome in the fully adjusted model (p for interaction = 0.48). Similarly, there was no significant interaction between PENK-A and sex for this outcome (p for interaction = 0.14).

In sensitivity analyses, baseline PENK-A was associated with incident CKD when using the less restrictive definition (OR: 1.13; 95% CI 1.01, 1.26), shown in Table 3.

**Table 3:** Association of PENK-A with incident CKD (defined as new eGFR < 60 mL/min/1.73m^2^ plus at least 25% eGFR decline)^a^

	**Quartiles of PENK-A**					
	**Q1**	**Q2**	**Q3**	**Q4**	**Per SD higher**	***P* value**^**+**^
	(OR, 95% CI)	(OR, 95% CI)	(OR, 95% CI)	(OR, 95% CI)	(OR, 95% CI)	
Range of PENK-A	< 48	48–56	57–70	> 70		
Number of events/Number at Risk (%)						
BioMedioR	112/909 (12.3)	135/908 (14.9)	172/909 (18.9)	205/908 (22.6)	624/3634 (17.2)	
Weighted	340/1760 (19.3)	395/1765 (22.4)	472/1756 (26.8)	516/1759 (29.3)	1723/7040 (24.5)	
Model 1*	Reference	1.17 (0.88, 1.53)	1.45 (1.11, 1.88)	1.87 (1.42, 2.45)	1.24 (1.13, 1.37)	< 0.001
Model 2**	Reference	1.40 (1.05, 1.87)	1.97 (1.49, 2.60)	2.93 (2.18, 3.94)	1.52 (1.35, 1.71)	< 0.001
Model 3***	Reference	1.12 (0.83, 1.51)	1.32 (0.98, 1.78)	1.43 (1.03, 1.97)	1.15 (1.01, 1.31)	0.03

P for interaction for sex: 0.18

P for interaction for race: 0.44

^a^Weighted to parent cohort and excluding participants missing PENK-A and baseline CKD

^*^Adjusted for age, sex, race

^**^Adjusted for Model 1 plus body mass index, current smoking, coronary artery disease, systolic blood pressure, use of antihypertensive meds, diabetes mellitus

^***^Adjusted for Model 2 plus baseline eGFR and uACR

^+^ *P* value for SD higher of PENK-A

### Relationship of PENK-A with progressive eGFR decline

Among all 7830 participants, an eGFR decline of ≥ 30% was observed in 1958 (25%) participants over 9.4 years. Higher PENK-A concentration was significantly associated with progressive eGFR decline across the sequence of adjusted models (OR for fully adjusted model: 1.11; 95% CI 1.00, 1.24) (Table 4). Once more, this association did not differ by race (p for interaction = 0.49) or sex (p for interaction = 0.11). This relationship appeared to increase generally monotonically across quartiles (Table 4).

**Table 4:** Association of PENK-A with progressive eGFR decline (greater or equal 30% decline from baseline)^a^

	**Quartiles of PENK-A**					
	**Q1**	**Q2**	**Q3**	**Q4**	**Per SD higher**	***P* value**^**+**^
	(OR, 95% CI)	(OR, 95% CI)	(OR, 95% CI)	(OR, 95% CI)	(OR, 95% CI)	
Range of PENK-A	< 48	48–58	59–72	> 72		
Number of events/Number at Risk (%)						
BioMedioR	192/979 (19.6)	189/979 (19.3)	217/979 (22.2)	233/978 (23.8)	831/3915 (21.2)	
Weighted	466/1961 (23.8)	462/1957 (23.6)	502/1957 (25.7)	528/1955 (27)	1958/7830 (25)	
Model 1^*^	Reference	0.95 (0.75, 1.19)	1.06 (0.84, 1.32)	1.09 (0.87, 1.37)	1.04 (0.96, 1.13)	0.38
Model 2^**^	Reference	1.08 (0.85, 1.36)	1.26 (1.00, 1.59)	1.34 (1.05, 1.70)	1.10 (1.01, 1.19)	0.03
Model 3^***^	Reference	1.08 (0.84, 1.37)	1.28 (1.00, 1.64)	1.32 (1.00, 1.75)	1.12 (1.00, 1.25)	0.05

P for interaction for sex: 0.11

P for interaction for race: 0.49

^a^Weighted to parent cohort and excluding participants missing PENK-A

^*^Adjusted for age, sex, race

^**^Adjusted for Model 1 plus body mass index, current smoking, coronary artery disease, systolic blood pressure, use of antihypertensive meds, diabetes mellitus

^***^Adjusted for Model 2 plus baseline eGFR and uACR

^+^ *P* value for SD higher of PENK-A

### Relationship of PENK-A with incident albuminuria

Among 5671 participants who did not have baseline albuminuria, 1279 (22.6%) had incident albuminuria at the second visit. In the final model, after adjustment for baseline eGFR and ACR, each SD higher of PENK-A was associated with incident albuminuria (OR: 1.16; 95% CI 1.02, 1.33) (Table 5). Once more, the increasing odds appeared generally monotonic across PENK-A quartiles.

**Table 5:** Association of PENK-A with incident albuminuria^a^

	**Quartiles of PENK-A**					
	**Q1**	**Q2**	**Q3**	**Q4**	**Per SD higher**	***P* value**^**+**^
	(OR, 95% CI)	(OR, 95% CI)	(OR, 95% CI)	(OR, 95% CI)	(OR, 95% CI)	
Range of PENK-A	< 48	48–58	59–72	> 72		
Number of events/Number at Risk (%)						
BioMedioR	122/826 (14.8)	124/869 (14.3)	162/877 (18.5)	186/829 (22.4)	594/3401 (17.5)	
Weighted	278/1422 (19.5)	275/1414 (19.4)	323/1418 (22.8)	403/1417 (28.4)	1279/5671 (22.6)	
Model 1^*^	Reference	0.92 (0.66, 1.29)	1.22 (0.89, 1.67)	1.70 (1.25, 2.31)	1.32 (1.17, 1.48)	< 0.001
Model 2^**^	Reference	1.00 (0.72, 1.41)	1.39 (1.01, 1.91)	1.98 (1.44, 2.73)	1.41 (1.24, 1.61)	< 0.001
Model 3^***^	Reference	0.96 (0.68, 1.36)	1.15 (0.81, 1.62)	1.31 (0.91, 1.90)	1.18 (1.02, 1.37)	0.03

P for interaction for sex: 0.97

P for interaction for race: 0.05

^a^Weighted to parent cohort and excluding participants missing PENK-A and baseline albuminuria

^*^Adjusted for age, sex, race

^**^Adjusted for Model 1 plus body mass index, current smoking, coronary artery disease, systolic blood pressure, use of antihypertensive meds, diabetes mellitus

^***^Adjusted for Model 2 plus baseline eGFR and uACR

^+^ *P* value for SD higher of PENK-A

The association of PENK-A with incident albuminuria differed by race (p for interaction = 0.05). Among White participants, the fully adjusted OR was 1.71 (95% CI 1.00, 2.95) as shown in Table 6, whereas the OR was 1.34 (95% CI 0.84, 2.14) among Black participants (Table 6). There was no interaction by sex (p for interaction = 0.97).

**Table 6:** Association of PENK-A with incident albuminuria stratified by race^a^

	**PENK-A**			
	**White Participants**		**Black Participants**	
	**Per SD higher**	***P*-value**	**Per SD higher**	***P*-value**
	(OR, 95% CI)		(OR, 95% CI)	
Number of events/Number at Risk (%)				
BioMedioR	291/1833 (15.9)		303/1568 (19.3)	
Weighted	670/3038 (22.1)		607/2633 (23.1)	
Model 1^*^	1.51 (1.27, 1.80)	< 0.001	1.15 (0.98, 1.36)	0.004
Model 2^**^	1.64 (1.35, 1.99)	< 0.001	1.24 (1.03, 1.48)	0.02
Model 3^***^	1.32 (1.06, 1.64)	0.02	1.05 (0.85, 1.29)	0.66

^a^Weighted to parent cohort and excluding participants missing PENK-A and baseline albuminuria

^*^Adjusted for age, sex

^**^Adjusted for Model 1 plus body mass index, current smoking, coronary artery disease, systolic blood pressure, use of antihypertensive meds, diabetes mellitus

^***^Adjusted for Model 2 plus baseline eGFR and uACR

We also found a significant interaction between PENK-A and diabetes mellitus (p for interaction = 0.03). In stratified analyses based on diabetes status, there was a significant association between PENK-A among those without diabetes mellitus (Table 7).

**Table 7:** Association of PENK-A with incident albuminuria stratified by diabetes status^a^

	**PENK-A**			
	**Without diabetes**		**With diabetes**	
	**Per SD higher**	***P*-value**	**Per SD higher**	***P*-value**
	(OR, 95% CI)		(OR, 95% CI)	
Number of events/Number at Risk (%)				
BioMedioR	285/2732 (10.4)		115/475 (24.2)	
Weighted	956/4584 (20.9)		652/1087 (60)	
Model 1^*^	1.51 (1.27, 1.80)	< 0.001	1.16 (0.92, 1.46)	0.20
Model 2^**^	1.53 (1.31, 1.79)	< 0.001	1.14 (0.90, 1.44)	0.27
Model 3^***^	1.29 (1.09, 1.53)	0.02	0.94 (0.70, 1.25)	0.67

P for interaction with diabetes mellitus: 0.03

^a^Weighted to parent cohort and excluding participants missing PENK-A and baseline albuminuria

^*^Adjusted for age, sex, race

^**^Adjusted for Model 1 plus body mass index, current smoking, coronary artery disease, systolic blood pressure, use of antihypertensive meds

^***^Adjusted for Model 2 plus baseline eGFR and uACR

## Discussion

In this biracial cohort of community-living participants of the REGARDS study, PENK-A concentration was associated with significant eGFR decline and incident albuminuria, but not incident CKD. The association with albuminuria was stronger in White compared to Black participants and only present among those without underlying diabetes mellitus.

Several prior studies evaluated the association between PENK-A concentration and incident CKD in other settings. In a population-based cohort in Sweden higher PENK-A concentration was associated with incident CKD, defined as an eGFR of < 60 ml/min/1.73m^2^ over 16.6 years using the CKD-EPI 2012 equation [16]. Kieneker et al. also noted an association between PENK-A and incident CKD among men but not women in the Prevention of Renal and Vascular End-stage Disease study (PREVEND), a population enriched with participants with albuminuria in the Netherlands [17].

Both prior studies were conducted among relatively homogenous European populations [16, 17]. The current study extends these findings to a biracial U.S. population. We found no association between PENK-A and incident CKD defined by eGFR < 60 ml/min/1.73m^2^ in individuals with baseline eGFR > 60 ml/min/1.73m^2^. Unlike the PREVEND report [17], there was no significant difference in associations by sex. Moreover, the association was similar irrespective of race suggesting that our inclusion of Black participants does not explain the disparate findings compared to prior research. We may not have found an association with incident CKD due to the strict CKD definition we used and the limited power in examining a binary outcome. However, we did find an association between higher baseline PENK-A concentration and progressive eGFR decline, which was similar by sex and race. Additionally, an association between PENK and the occurrence of CKD was found when a 25% rather than 40% decrease in eGFR was used in conjunction with a new eGFR of less than 60 mL/min/1.73 m^2^.

PENK-A is a robust filtration marker compared to creatinine [15]. Donato et al. studied the relationship between plasma PENK-A concentration and measured GFR (mGFR) assessed by iothalamate among patients with and without kidney disease [15]. They found a stronger association between PENK-A and mGFR than creatinine. Thus, the associations of PENK-A with significant eGFR decline, even after adjusting for eGFR, may simply reflect that PENK-A may be a more reliable biomarker of mGFR than creatinine. In our study, PENK-A was more strongly correlated with cystatin-C than with serum creatinine. A prior genome-wide association analysis among White persons found that genetic variation at the PENK-A locus was associated with higher pro-ENK levels [16]. Moreover, Mendelian randomization analysis suggested that PENK-A may have a causal role in incident CKD [16]. Putative mechanisms to support this hypothesis are unknown and require additional study.

Above and beyond longitudinal declines in eGFR, albuminuria is an early marker of kidney disease [25]. It represents a potential risk factor for kidney failure irrespective of the presence or absence of diabetes [26, 27]. In the present study, PENK-A was associated with incident albuminuria; an association that was particularly strong in White participants. We did not find an association among those without diabetes, but we attribute this to the sample size among those with diabetes and the underlying albuminuria in this group. To our knowledge, only one previous study addressed this question; Kieneker et al. found that PENK-A was associated with albuminuria, graft failure, and mortality among kidney transplant recipients [28]. However, when the authors evaluated this outcome among participants without kidney transplants, there was no association [17]. Differences in study populations may explain the disparate findings, as our study population was older, had higher BMI, higher SBP, higher prevalence of diabetes, and lower eGFR compared to the study by Kieneker et al. [17]. Since albuminuria is such a potent risk marker for CKD progression, this finding deserves further investigation. We also noted a stronger association between PENK-A and incident albuminuria among White participants than their Black counterparts. We suspect that the finding in our study was due to a higher number of Black participants who were excluded from the analyses because of albuminuria ≥ 30 mg/g at baseline. We excluded 16.9% of Blacks participants and 9.7% of Whites for the final analysis due to presence of albuminuria at baseline.

Strengths of this research include evaluation of a well-characterized cohort that was intentionally selected to evaluate differences in health between White and Black adults and males and females from regions of the U.S. with a high prevalence of diabetes and hypertension. We also used a strict definition of incident CKD and had the opportunity to examine eGFR decline and incident albuminuria. A wide array of traditional CKD risk factors were robustly measured at baseline to allow evaluation of confounding.

Our study also has important limitations. We only measured PENK-A at baseline, so we could not evaluate longitudinal change. Estimated GFR and albuminuria values were only collected at two-time points ten years apart, so we cannot assess shorter-term changes and address questions of informative dropout due to death or illness. However, the BioMedioR design assured nearly complete data on kidney disease at the two-time points allowing us to evaluate the outcomes of interest. Additionally, prior studies showed no impact of informative missingness in analyzing race differences in other studies in REGARDS [29, 30].

In conclusion, in a biracial cohort, higher PENK-A concentration was associated with a higher incidence of significant decline of eGFR overall, although associations with incident CKD depended upon the definition. PENK-A was also associated with a higher incidence of albuminuria, an association that was stronger in White than in Black participants and only among those without diabetes mellitus. Future studies are warranted to confirm these results and evaluate if PENK-A may be incorporated in the risk assessment for the development of CKD.

## Data Availability

All data generated or analyzed during this study are included in this published article.

## References

[1] Levey AS, Stevens LA, Schmid CH (2009). A new equation to estimate glomerular filtration rate. Ann Intern Med.

[2] Tonelli M, Wiebe N, Culleton B (2006). Chronic kidney disease and mortality risk: a systematic review. J Am Soc Nephrol.

[3] Golestaneh L, Alvarez PJ, Reaven NL (2017). All-cause costs increase exponentially with increased chronic kidney disease stage. Am J Manag Care.

[4] Kassirer JP (1971). Clinical evaluation of kidney function–glomerular function. N Engl J Med.

[5] Coresh J, Selvin E, Stevens LA (2007). Prevalence of chronic kidney disease in the United States. JAMA.

[6] de Jong PE, Gansevoort RT (2008). Fact or fiction of the epidemic of chronic kidney disease–let us not squabble about estimated GFR only, but also focus on albuminuria. Nephrol Dial Transplant.

[7] Grossman A, Clement-Jones V (1983). Opiate receptors: enkephalins and endorphins. Clin Endocrinol Metab.

[8] Beunders R, Struck J, Wu AHB (2017). Proenkephalin (PENK) as a Novel Biomarker for Kidney Function. J Appl Lab Med.

[9] Denning GM, Ackermann LW, Barna TJ, Armstrong JG, Stoll LL, Weintraub NL, Dickson EW (2008). Proenkephalin expression and enkephalin release are widely observed in non-neuronal tissues. Peptides.

[10] Grossman A, Besser GM, Milles JJ, Baylis PH (1980). Inhibition of vasopressin release in man by an opiate peptide. Lancet (London, England).

[11] Sezen SF, Kenigs VA, Kapusta DR (1998). Renal excretory responses produced by the delta opioid agonist, BW373U86, in conscious rats. J Pharmacol Exp Ther.

[12] Mosnaim AD, Puente J, Wolf ME, Callaghan OH, Busch R, Diamond S (1988). Studies of the in vitro human plasma degradation of methionine-enkephalin. Gen Pharmacol.

[13] Mosnaim AD, Puente J, Saavedra R, Diamond S, Wolf ME (2003). In vitro human plasma leucine(5)-enkephalin degradation is inhibited by a select number of drugs with the phenothiazine molecule in their chemical structure. Pharmacology.

[14] Ernst A, Köhrle J, Bergmann A (2006). Proenkephalin A 119–159, a stable proenkephalin A precursor fragment identified in human circulation. Peptides.

[15] Donato LJ, Meeusen JW, Lieske JC, Bergmann D, Sparwasser A, Jaffe AS (2018). Analytical performance of an immunoassay to measure proenkephalin. Clin Biochem.

[16] Schulz CA, Christensson A, Ericson U (2017). High level of fasting plasma proenkephalin-a predicts deterioration of kidney function and incidence of CKD. J Am Soc Nephrol.

[17] Kieneker LM, Hartmann O, Bergmann A (2018). Proenkephalin and risk of developing chronic kidney disease: the prevention of renal and vascular end-stage disease study. Biomarkers.

[18] Daneshpajouhnejad P, Kopp JB, Winkler CA, Rosenberg AZ (2022). The evolving story of apolipoprotein L1 nephropathy: the end of the beginning. Nat Rev Nephrol.

[19] Howard VJ, Cushman M, Pulley L (2005). The reasons for geographic and racial differences in stroke study: objectives and design. Neuroepidemiology.

[20] Warnock DG, McClellan W, McClure LA (2005). Prevalence of chronic kidney disease and anemia among participants in the Reasons for Geographic and Racial Differences in Stroke (REGARDS) Cohort Study: baseline results. Kidney Int.

[21] Long DL, Guo B, McClure LA (2022). Biomarkers as MEDiators of racial disparities in risk factors (BioMedioR): Rationale, study design, and statistical considerations. Ann Epidemiol.

[22] Gillett SR, Boyle RH, Zakai NA, McClure LA, Jenny NS, Cushman M (2014). Validating laboratory results in a national observational cohort study without field centers: the reasons for geographic and racial differences in stroke cohort. Clin Biochem.

[23] Bash LD, Coresh J, Köttgen A, Parekh RS, Fulop T, Wang Y, Astor BC (2009). Defining incident chronic kidney disease in the research setting: The ARIC Study. Am J Epidemiol.

[24] Inker LA, Eneanya ND, Coresh J (2021). New Creatinine- and Cystatin C-Based Equations to Estimate GFR without Race. N Engl J Med.

[25] de Jong PE, Curhan GC (2006). Screening, monitoring, and treatment of albuminuria: Public health perspectives. J Am Soc Nephrol.

[26] Nelson RG, Bennett PH, Beck GJ (1996). Development and progression of renal disease in Pima Indians with non-insulin-dependent diabetes mellitus diabetic renal disease study group. New England J Med.

[27] Verhave JC, Gansevoort RT, Hillege HL, Bakker SJ, De Zeeuw D, de Jong PE (2004). An elevated urinary albumin excretion predicts de novo development of renal function impairment in the general population. Kidney Int Suppl.

[28] Kieneker LM, Hartmann O, Struck J (2017). Plasma proenkephalin and poor long-term outcome in renal transplant recipients. Transplantation direct.

[29] Howard G, Lackland DT, Kleindorfer DO (2013). Racial differences in the impact of elevated systolic blood pressure on stroke risk. JAMA Intern Med.

[30] Long DL, Howard G, Long DM (2019). An investigation of selection bias in estimating racial disparity in stroke risk factors. Am J Epidemiol.

